# Supplementary material for: Adsorption of Reactive Blue 116 Dye and Reactive Yellow 81 Dye from Aqueous Solutions by Multi-Walled Carbon Nanotubes
Source: Materials (Basel). 2020 Jun 18;13(12):2757. doi: 10.3390/ma13122757 (PMC7345466; doi:10.3390/ma13122757)
Supplement: Supplementary file 1 [file materials-13-02757-s001.pdf]

materials

Supplementary Materials: Adsorption of Reactive Blue 116 Dye and Reactive Yellow 81 Dye from Aqueous Solutions by Multi-Walled Carbon Nanotubes

Christian De Benedetto, Anastasia Macario, Carlo Siciliano, Jànos B.Nagy and Pierantonio De Luca *

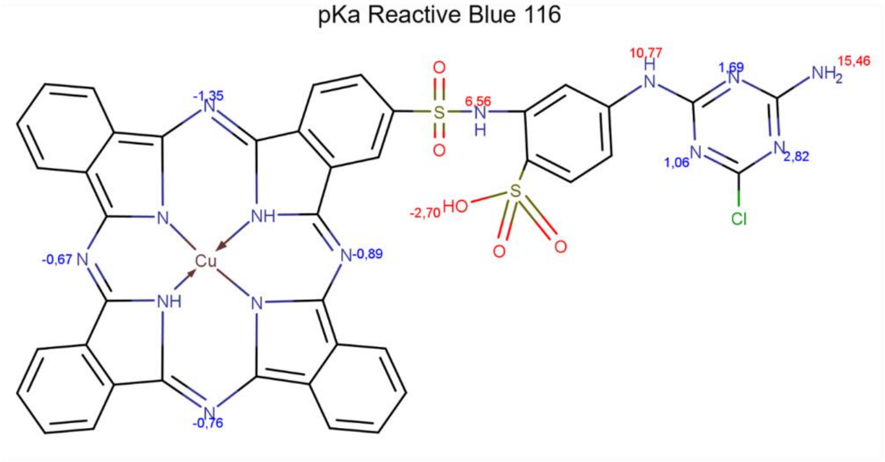

(a)

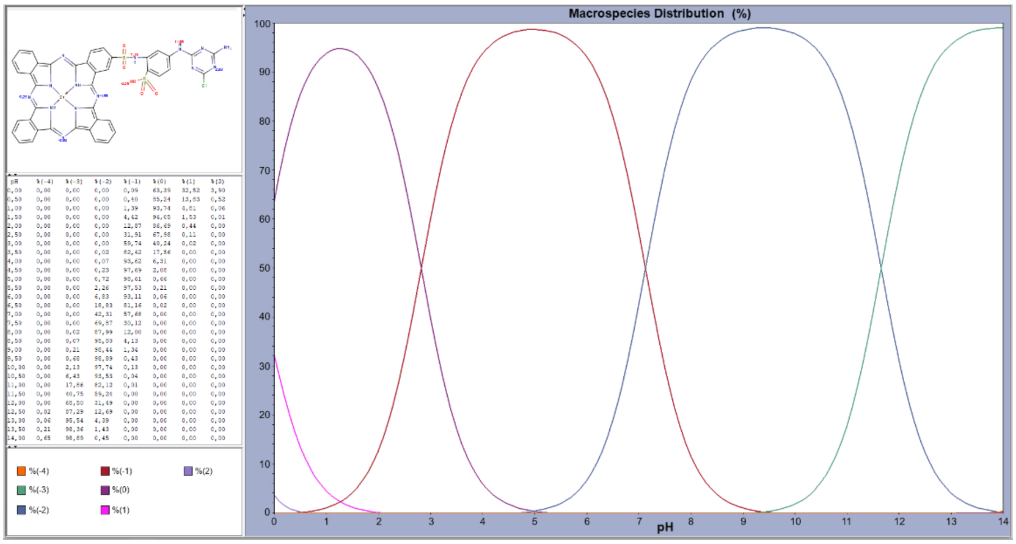

(b)

Figure S1. (**a**) RB-116 pKa protonation mechanism; (**b**) Formation and distribution diagram of the RB-116 complex related on the pH.

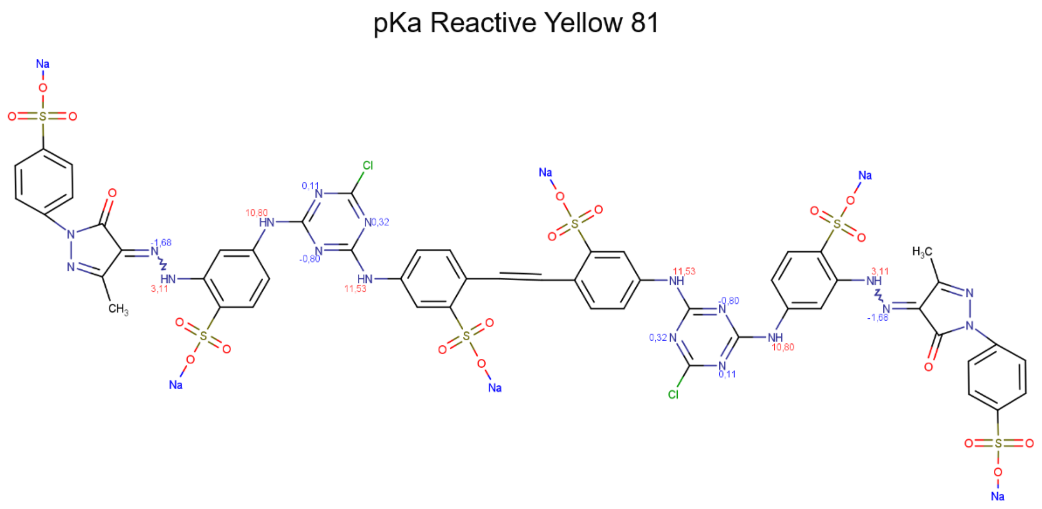

(a)

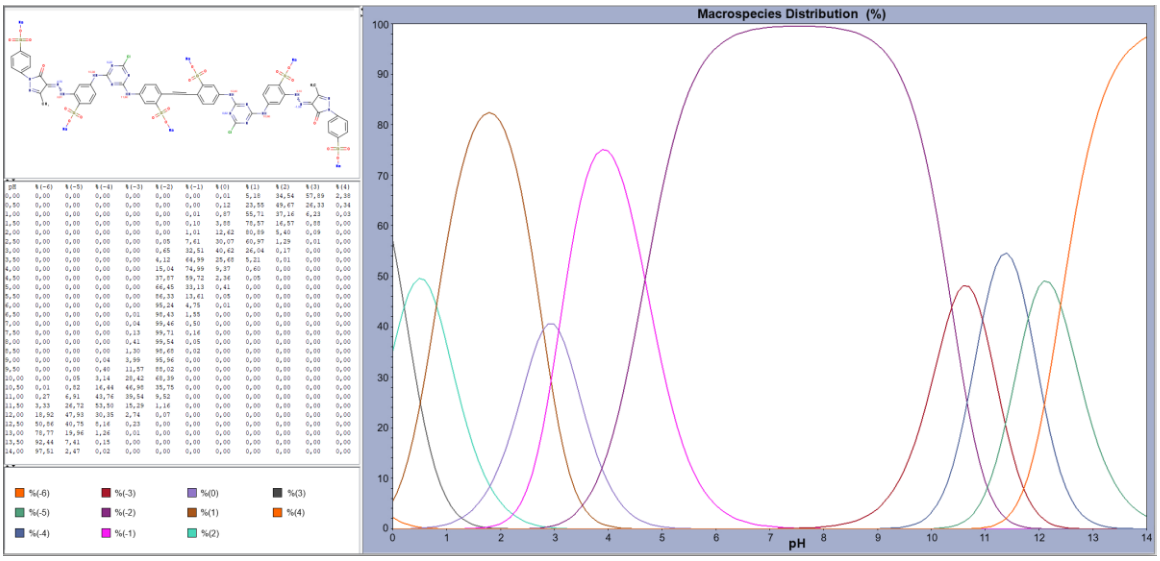

pH	%(-6)	%(-5)	%(-4)	%(-3)	%(-2)	%(-1)	%(0)	%(1)	%(2)	%(3)	%(4)
0,00	0,00	0,00	0,00	0,00	0,00	0,00	0,01	5,18	34,54	57,89	2,38
0,50	0,00	0,00	0,00	0,00	0,00	0,00	0,12	23,55	49,67	26,33	0,34
1,00	0,00	0,00	0,00	0,00	0,00	0,01	0,87	55,71	37,16	6,23	0,03
1,50	0,00	0,00	0,00	0,00	0,00	0,10	3,88	78,57	16,57	0,88	0,00
2,00	0,00	0,00	0,00	0,00	0,00	1,01	12,62	80,89	5,40	0,09	0,00
2,50	0,00	0,00	0,00	0,00	0,05	7,61	30,07	60,97	1,29	0,01	0,00
3,00	0,00	0,00	0,00	0,00	0,65	32,51	40,62	26,04	0,17	0,00	0,00
3,50	0,00	0,00	0,00	0,00	4,12	64,99	25,68	5,21	0,01	0,00	0,00
4,00	0,00	0,00	0,00	0,00	15,04	74,99	9,37	0,60	0,00	0,00	0,00
4,50	0,00	0,00	0,00	0,00	37,87	59,72	2,36	0,05	0,00	0,00	0,00
5,00	0,00	0,00	0,00	0,00	66,45	33,13	0,41	0,00	0,00	0,00	0,00
5,50	0,00	0,00	0,00	0,00	86,33	13,61	0,05	0,00	0,00	0,00	0,00
6,00	0,00	0,00	0,00	0,00	95,24	4,75	0,01	0,00	0,00	0,00	0,00
6,50	0,00	0,00	0,00	0,01	98,43	1,55	0,00	0,00	0,00	0,00	0,00
7,00	0,00	0,00	0,00	0,04	99,46	0,50	0,00	0,00	0,00	0,00	0,00
7,50	0,00	0,00	0,00	0,13	99,71	0,16	0,00	0,00	0,00	0,00	0,00
8,00	0,00	0,00	0,00	0,41	99,54	0,05	0,00	0,00	0,00	0,00	0,00
8,50	0,00	0,00	0,00	1,30	98,68	0,02	0,00	0,00	0,00	0,00	0,00
9,00	0,00	0,00	0,04	3,99	95,96	0,00	0,00	0,00	0,00	0,00	0,00
9,50	0,00	0,00	0,40	11,57	88,02	0,00	0,00	0,00	0,00	0,00	0,00
10,00	0,00	0,05	3,14	28,42	68,39	0,00	0,00	0,00	0,00	0,00	0,00
10,50	0,01	0,82	16,44	46,98	35,75	0,00	0,00	0,00	0,00	0,00	0,00
11,00	0,27	6,91	43,76	39,54	9,52	0,00	0,00	0,00	0,00	0,00	0,00
11,50	3,33	26,72	53,50	15,29	1,16	0,00	0,00	0,00	0,00	0,00	0,00
12,00	18,92	47,93	30,35	2,74	0,07	0,00	0,00	0,00	0,00	0,00	0,00
12,50	50,86	40,75	8,16	0,23	0,00	0,00	0,00	0,00	0,00	0,00	0,00
13,00	78,77	19,96	1,26	0,01	0,00	0,00	0,00	0,00	0,00	0,00	0,00
13,50	92,44	7,41	0,15	0,00	0,00	0,00	0,00	0,00	0,00	0,00	0,00
14,00	97,51	2,47	0,02	0,00	0,00	0,00	0,00	0,00	0,00	0,00	0,00

(b)

Figure S2. (**a**) RY-81 pKa protonation mechanism; (**b**) Formation and distribution diagram of the RY-81 complex related on the pH.

© 2020 by the authors. Licensee MDPI, Basel, Switzerland. This article is an open access article distributed under the terms and conditions of the Creative Commons Attribution (CC BY) license (http://creativecommons.org/licenses/by/4.0/).
